# Immediate implants placed in fresh sockets associated to periapical infectious processes. A systematic review

**DOI:** 10.4317/medoral.18942

**Published:** 2013-05-31

**Authors:** Juan C. Álvarez-Camino, Eduard Valmaseda-Castellón, Cosme Gay-Escoda

**Affiliations:** 1 DDS. Resident of the Master of Oral Surgery and Implantology. University of Barcelona Dental School; 2 DDS, PhD, MS, EBOS. Professor of Oral Surgery. Professor of the Master of Oral Surgery and Implantology. Barcelona University Dental School. Researcher of the IDIBELL Institute; 3 MD, DDS, PhD, MS, EBOS. Chairman and Professor of Oral and Maxillofacial Surgery. Director of the Master of Oral Surgery and Implantology. School of Dentistry of the University of Barcelona. Coordinator/Researcher of the IDIBELL Institute. Head of the Oral and Maxillofacial Surgery Department of the Teknon Medical Center, Barcelona, Spain

## Abstract

Objetives: The development of treated implant surfaces, added to the increase of the aesthetic requirements by the patients has led to a change in the treatment protocols as well as the development of techniques such as the one-fase implants and the immediate prosthetic loading. One of the usual contraindications of the implant treatment is the presence of periapical disease associated to the tooth to be replaced. The aim of this paper is to review the published literature on immediate implant placement in extraction sockets of teeth with periapical pathology, considering the level of scientific evidence, and following the principles of medicine and evidence-based Dentistry. Material and Methods: A search of articles published between 1982 and 2012 was conducted. The search terms immediate, dental implant, extraction, infected, periapical pathology were used. Search was limited to studies in animals and humans, published in english language. Results: 16 articles were selected from a total of 438, which were stratified according to their level of scientific evidence using the SORT criteria (Strength of Recommendation Taxonomy). Studies in both animals and humans presented high rates of implant survival, but human studies are limited to a small number of cases. Discussion and Conclusions: There is a limited evidence regarding implant placement immediately to the extraction of teeth affected by chronic periapical pathology. Following analysis of the articles, and in function of their scientific quality, a type B recommendation is given in favor of the immediate implant placement in fresh sockets associated to periapical infectious processes.

** Key words:**Immediate implant, periapical pathology.

## Introduction

The first dental implant protocol presented by Brånemark et al. (1) included a two-stage surgical procedure, separated by a period of osteointegration of six months as minimum, prior to the prosthetic loading of the implant at the mandible. This, added to the period of wound healing and postextraction bone formation, was invariably associated with aesthetics periodontal alterations due to the localized bone resorption observed at the extraction area, caused by the absence of the stimulus associated to the periodontal ligament, as well as the remodeling of the soft tissues (2), despite the clinical success demonstrated in many cases.

Residual bone volume could be reduced significantly because of the alveolar bone resorption associated to tooth extraction, compromising the subsequent implant treatment, hindering the implant placement in a favorable position, a necessary step for a proper prosthetic restoration (3). This situation is more evident in the anterior maxilla, where resorption of bone tissue can force to place the implant in a palatal position, which compromises the prosthetic result (3).

The esthetic requirements, as well as the patient needs have brought changes to the implant protocols. The use of treated surfaces implants has allowed for more freedom in the selection of the socket as implant recipients, as well as the development of surgical techniques which make possible the reduction of the treatment period. All this, to give the patient an optimal aesthetic solution almost immediately, provided that the respect of the specific protocol for proper primary stability is observed.

The immediate implant placement in extraction site is a treatment with a defined protocol, and well accepted, thanks to the preservation of aesthetics, the maintenance of the alveolar walls, a better positioning of the implant, and a reduction in surgery time and the overall treatment (4). However, the concept of immediate implant placement after extraction of a tooth with periapical disease is a very controversial topic, with few scientific studies of quality published.

Many authors have suggested that the immediate implant placement in a socket with the presence of infectious disease would be completely contraindicated (5,6), because contamination could compromise the osseointegration process. Alsaadi et al. (7) in a case-control study, reported a greater loss of implants in those sockets with periapical lesions, especially when machined surface implants were placed. An increased loss of endosseous implants also has been associated with periodontal disease (2,7-9). However, recent researches show satisfactory results in the immediate implant placement in sockets with chronic periapical disease (3,9).

Moreover, in the vertebral osteomyelitis, a meticulous debridement of bone, joined by a strong antibiotic therapy, prior to use of a titanium cage as a provider of immediate support and stability for weakened vertebrae have provided satisfactory results (10). It is posibble to obtain a correct osseointegration between titanium structures and bone, despite substantial previous infectious process (10). Authors like Naves et al. (9) state that these results can be considered equivalent to the osseointegration of endosseous oral implants.

In addition, recent animal studies have shown that by proper debridement and prophylactic use of antibiotics it is possible to create adequate local conditions to produce a bone remodeling process around the immediately dental implant placed in a socket associated with infectious disease (11).

The aim of this paper is to identify the articles published on the placement of immediate implant in extraction sockets of teeth with periapical pathology, as well as to classify these papers according to their level of scientific evidence, using the SORT criteria (Strength of Recommendation Taxonomy).

## Material and Methods

A PubMed-MEDLINE and Cochrane databases search of articles published between 1982 and 2012 was conducted during May 2012. In an initial search, the terms *“immediate”, “dental implant”, “extraction”, “infected”, “periapical”, “pathology”* were used. Search was limited to animal and human studies, and articles written in English. The terms were then merged in a second search, using the Boolean operator “AND”, in order to obtain the articles that included two or more of the used search terms. Items found were analyzed to verify the relevance of these in relation to the topic under study. The irrelevant articles were discarded. Next, the items were stratified according to their level of scientific evidence, using the SORT criteria (Strength of Recommendation Taxonomy) ( Table 1, Table 2) Only articles classified on the firsts two levels were selected. Subsequently, according to the level of scientific evidence of the articles reviewed, a recommendation level was declared in favor of, or against the use of immediate osseointegrated implants in sockets associated to periapical infectious processes.

**Table 1:**
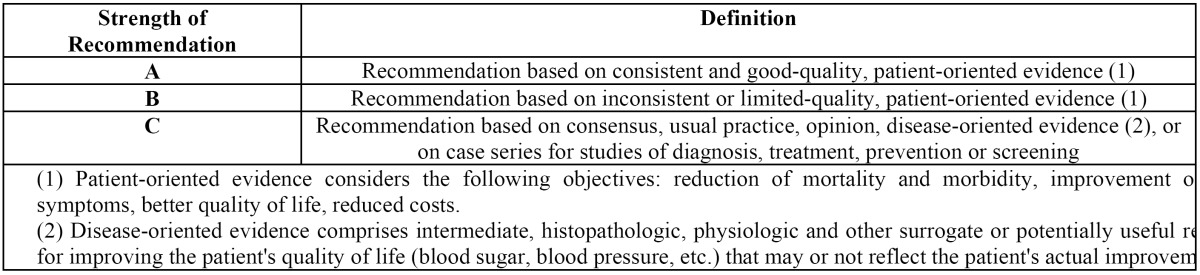
SORT Criteria (Strength of Recommendation Taxonomy).

**Table 2:**
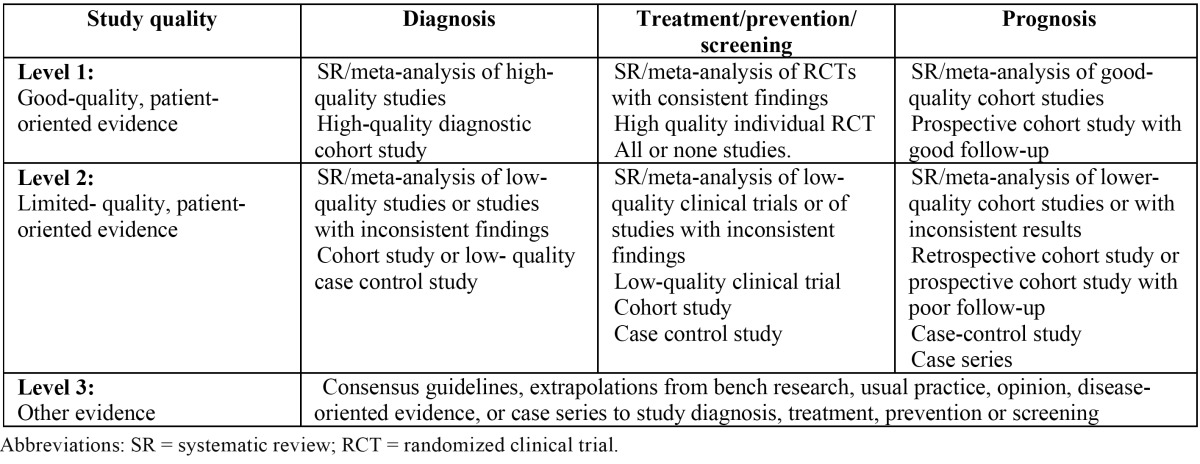
Levels of scientific evidence.

## Results

**Figure 1:**
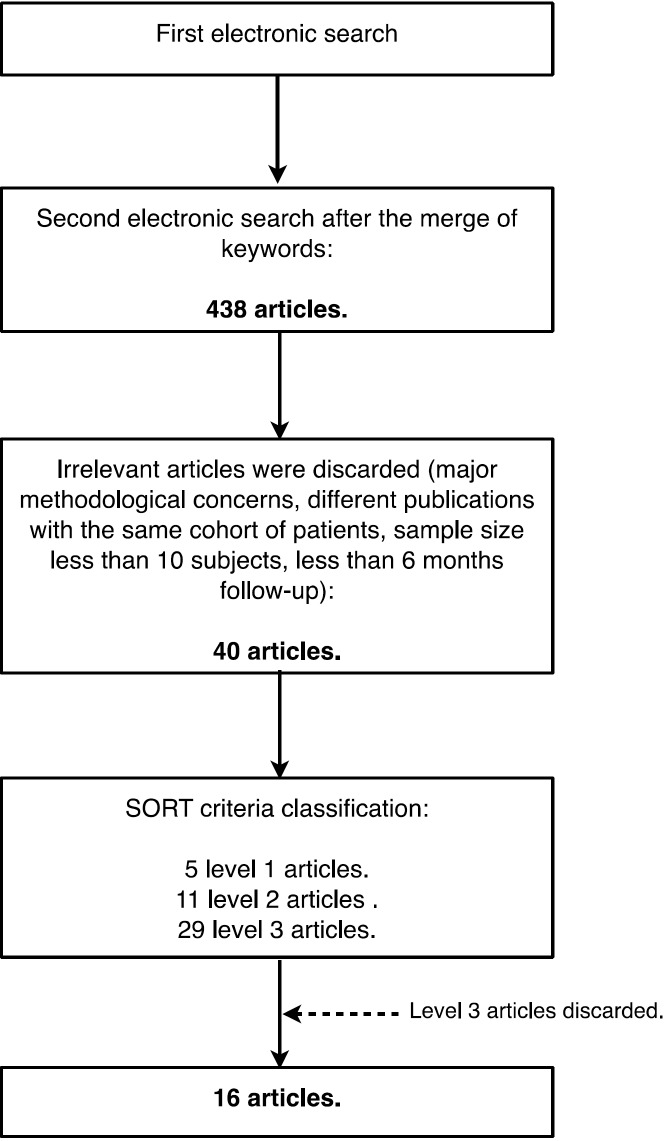
Search strategy.

The initial search conducted in the PubMed-MEDLINE database provided a total of 142,866 articles for the term “immediate” 24,706 articles for “dental implant”, 164,095 for the term “extraction”, 288,205 for the term “infected”, 8,150 articles for the term “periapical” and 2,242,481 for “pathology”. After a second electronic search, which merged keywords, 438 articles were obtained which showed two or more of the terms used. As for the Cochrane Library database search, it provided with 8,942 articles to the term “immediate”, 1,154 articles for “dental implant”, 6,234 for the term “extraction”, 6,550 for the term “infected”, 279 for the term “periapical” and 32,024 for the term “pathology” (Fig. 1).

In the first instance, irrelevant articles and those who had significant methodological errors, absence of criteria for sample selection, different publications with the same cohort of patients, a loose definition of the study groups or a period follow-up less than six months were discarded.

Following this initial analysis, a total of 42 articles with relevance to our review were obtained. These items were stratified by level of scientific evidence, using the SORT criteria. A total of 16 items were obtained, 5 of with a level of scientific evidence of 1 and 11 with a scientific evidence level of 2 ( Table 3). Items with a level of evidence 3 were discarded.

**Table 3:**
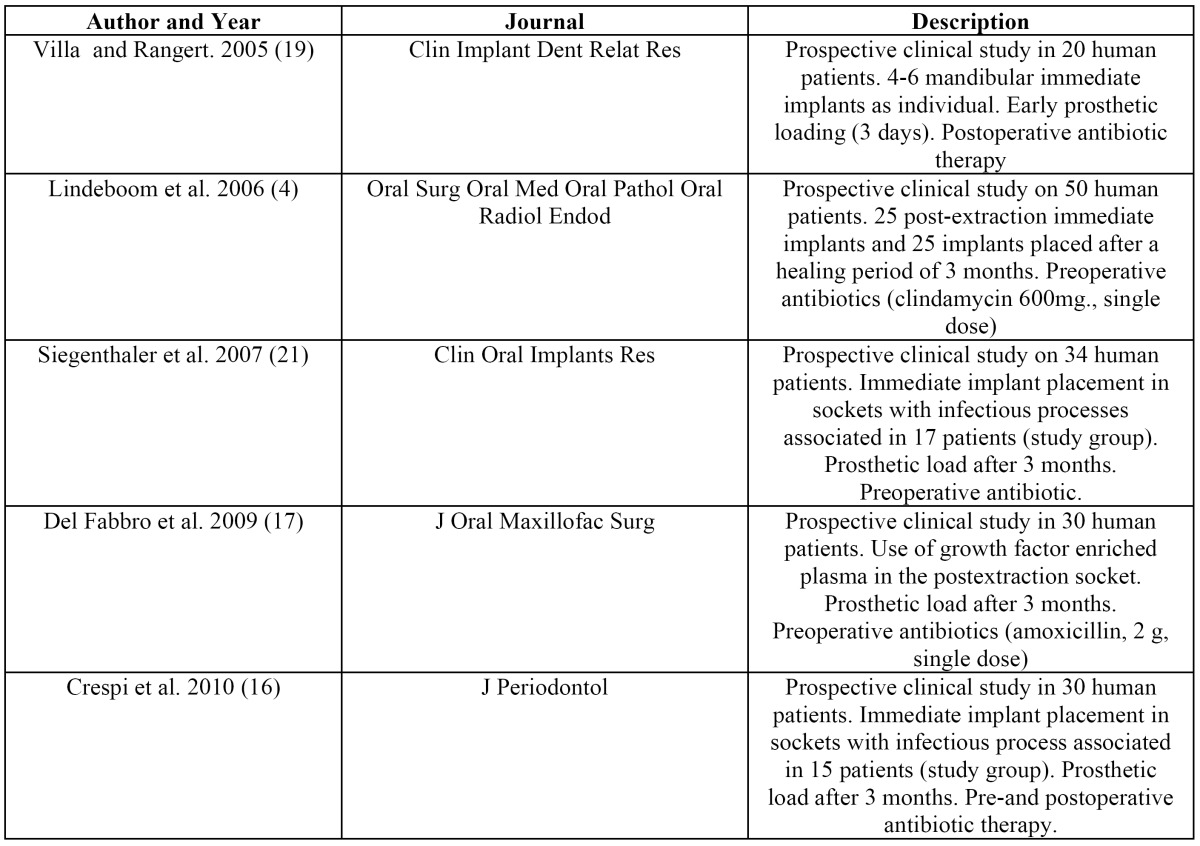
Level 1 studies that analize the immediate placement of implant in sockets associated with periapical infectious process.

## Discussion

Several authors have proposed the immediate implant placement in extraction sockets to reduce the alveolar bone resorption process and to minimize the time of implant treatment (12). The immediate post-extraction implant placement has success rates similar to those obtained when the implant is placed on a deferred basis (13). However, there are few clinical data regarding the immediate implant placement in sockets associated with chronic periapical infectious processes.

Some clinical studies have suggested that a history of periodontal disease and periapical infection could be used as a predictive marker of peri-implant disease, as well as implant failure (14,15) and therefore discourage the placement of implants in the presence of periapical and periodontal pathology. The reason is that there might be a potential contamination of the implant during the initial phase of wound and bone healing due to the remnant of infection, which affects the osseointegration process (15).

Most of the authors agree that, while there is a remnant of the correct architecture extraction site, the prognosis of the implant will be good in most cases (4,16,17). Atraumatic extraction of the affected tooth, using wide platform implants and guided bone regeneration (GBR) techniques can significantly improve the prognosis of the case (17).

Back in 1995, Novaes and Novaes Jr. (11) defended the possibility of the placement of immediate implants if sockets associated with chronic periapical infection, as long as a proper postoperative antibiotic coverage is performed. In a histomorphometric study in dogs, Novaes et al. (18) demonstrated that the osseointegration levels in those immediate implants in fresh extraction sockets associated with infection (study group) did not differ significantly from those implants in healthy sockets.

In studies such as Crespi et al. (16) the marginal bone level in those immediate implants in sockets with a history of infection remained at levels similar to those with healthy socket implants. Furthermore, an increase in the levels of peri-implant bone mineralization after 48 months is described as well. Similar results were obtained by Villa and Rangert (19,20), who evaluated the survival rates of immediate implants after extraction of teeth with periodontal and endodontic lesions followed by curettage of the apical socket and irrigation with antibiotic solution. No signs of implant-associated local infection were detected after one year. These positive results could be explained by various biological events occurring during bone healing process, dependent on aspects such as primary stability of the implant, the surgical technique, the prosthetic load and the associated inflammatory response.

In human studies (16,19-21) the implant placement was performed after the extraction of teeth with sigs of chronic periapical periodontitis, presence of radiolucent periapical images, presence of fistula and purulent discharge (20,21). In these studies (20,21) a mucoperiosteal flap was raised, the apical granulation tissue was removed, and then the socket was irrigated with sterile saline. This procedure is not associated with an increased presence of postoperative complications in those implants which achieved a good primary stability. The authors conclude that the extraction of the affected tooth and the curettage of the alveolar socket led to the elimination of the associated infection, and the immediate implant placement contributed to the maintaince of the alveolar bone architecture, as well as the preservation of the interdental papilla around the implant-supported restorations. Crespi et al. (16) explained the high success rate of immediate implants in sockets with presence of chronic and acute infections through the endoperiodontal origin of the infection, associated with anaerobic bacteria (Fusobacterium, Prevotella, Porphyromonas, Actinomyces, Streptococcus, Peptostreptococcus), and the variations in the anaerobic environment that occur after the extraction and curettage of the socket, which would lead to the eradication of the disease-associated endoperiodontal microbiota.

One aspect in which there is some disagreement among authors who advocate this technique would be in use of antibiotic medication before and after the implant surgery. Both Lindeboom et al. (4) as Siegenthaler et al. (21) included in their surgical protocol the use of preoperative antibiotics (clindamycin 600 mg, one hour before surgery), while Casap et al. (3) indicate the preoperative use of a daily dose of 1.5 g of amoxicillin four days prior to surgery, maintaining the same dose for ten days during the postoperative course; This authors describe a case of pseudomembranous colitis as a postoperative complication, associated the chronic use of antibiotics. The rest of the studies reviewed did not include within their protocol the use of antibiotic premedication, although authors like Novaes and Novaes Jr. (11), Villa and Rangert (19) and Siegenthaler et al. (21) recommend the use of postoperative antibiotics, in different doses and for different time periods, with no consensus.

## Conclusions

Being a controversial procedure, and with a little scientific literature that addresses this issue, it is very difficult to state categorically that immediate implant placement in sockets associated to endoperiodontal infection can be considered a reliable treatment. Moreover, there is disagreement on what should be the surgical protocol, and the indication of antibiotic therapy prior to surgery. Following analysis of the articles, and in function of their scientific quality, a type B recommendation is given in favor of the immediate implant placement in fresh sockets associated to periapical infectious processes.

## References

[1] BrÃnemark PI, Hansson BO, Adell R, Breine U, LindstrÃm J, HallÃn O (1977). Osseointegrated implants in the treatment of the edentulous jaw. Experience from a 10-year period. Scand J Plast Reconstr Surg Suppl.

[2] Cardaropoli G, AraÃjo M, Hayacibara R, Sukekava F, Lindhe J (2005). Healing of extraction sockets and surgically produced - augmented and non-augmented - defects in the alveolar ridge. An experimental study in the dog. J Clin Periodontol.

[3] Casap N, Zeltser C, Wexler A, Tarazi E, Zeltser R (2007). Immediate placement of dental implants into debrided infected dentoalveolar sockets. J Oral Maxillofac Surg.

[4] Lindeboom JAH, Tjiook Y, Kroon FHM (2006). Immediate placement of implants in periapical infected sites: A prospective randomized study in 50 patients. Oral Surg Oral Med Oral Pathol Oral Radiol Endod.

[5] Schwartz-Arad D, Chaushu G (1997). The ways and wherefores of immediate placement of implants into fresh extraction sites: A literature review. J Periodontol.

[6] Becker W, Becker BE (1990). Guided tissue regeneration for implants placed into extraction sockets and for implant dehiscences: Surgical techniques and case report. Int J Periodontics Restorative Dent.

[7] Alsaadi G, Quirynen M, KomÃrek A, van Steenberghe D (2007). Impact of local and systemic factors on the incidence of oral implant failures, up to abutment connection. J Clin Periodontol.

[8] Evian CI, Emling R, Rosenberg ES, Waasdorp JA, Halpern W, Shah S (2004). Retrospective analysis of implant survival and the influence of periodontal disease and immediate placement on long-term results. Int J Oral Maxillofac Implants.

[9] Naves MM, Horbylon BZ, Gomes CF, Menezes HHM, Bataglion C, MagalhÃes D (2009). Immediate implants placed into infected sockets: a case report with 3-year follow-up. Braz Dent J.

[10] Liljenqvist U, Lerner T, Bullmann V, Hackenberg L, Halm H, Winkelmann W (2003). Titanium cages in the surgical treatment of severe vertebral osteomyelitis. Eur Spine J.

[11] Novaes AB Jr, Novaes AB (1995). Immediate implants placed into infected sites: A clinical report. Int J Oral Maxillofac Implants.

[12] Lazzara RJ (1989). Immediate implant placement into extraction sites: surgical and restorative advantages. Int J Periodontics Restorative Dent.

[13] Chen ST, Wilson TG, HÃmmerle CHF (2004). Immediate or early placement of implants following tooth extraction: Review of biologic basis, clinical procedures, and outcomes. Int J Oral Maxillofac Implants.

[14] Ayangco L, Sheridan PJ (2001). Development and treatment of retrograde peri-implantitis involving a site with a history of failed endodontic and apicoectomy procedures: A series of reports. Int J Oral Maxillofac Implants.

[15] Karoussis IK, Salvi GE, Heitz-Mayfield LJA, BrÃgger U, HÃmmerle CHF, Lang NP (2003). Long-term implant prognosis in patients with and without a history of chronic periodontitis: A 10-year prospective cohort study of the ITI Dental Implant System. Clin Oral Implants Res.

[16] Crespi R, CapparÃ P, Gherlone E (2010). Fresh-socket implants in periapical infected sites in humans. J Periodontol.

[17] Del Fabbro M, Boggian C, Taschieri S (2009). Immediate implant placement into fresh extraction sites with chronic periapical pathologic features combined with plasma rich in growth factors: Preliminary results of single-cohort study. J Oral Maxillofac Surg.

[18] Novaes AB, Vidigal JÃnior GM, Novaes AB, Grisi MF, Polloni S, Rosa A (1998). Immediate implants placed into infected sites: A histomorphometric study in dogs. Int J Oral Maxillofac Implants.

[19] Villa R, Rangert B (2005). Early loading of interforaminal implants immediately installed after extraction of teeth presenting endodontic and periodontal lesions. Clin Implant Dent Relat Res.

[20] Villa R, Rangert B (2007). Immediate and early function of implants placed in extraction sockets of maxillary infected teeth: A pilot study. J Prosthet Dent.

[21] Siegenthaler DW, Jung RE, Holderegger C, Roos M, HÃmmerle CHF (2007). Replacement of teeth exhibiting periapical pathology by immediate implants: A prospective, controlled clinical trial. Clin Oral Implants Res.

[22] Novaes AB Jr, Marcaccini AM, Souza SL, Taba M Jr (2003). , Grisi MF. Immediate placement of implants into periodontally infected sites in dogs: A histomorphometric study of bone-implant contact. Int J Oral Maxillofac Implants.

[23] Marcaccini AM, Novaes AB Jr, Souza SL, Taba M Jr (2003). ,Grisi MF. Immediate placement of implants into periodontally infected sites in dogs. Part 2: A fluorescence microscopy study. Int J Oral Maxillofac Implants.

[24] Chang SW, Shin SY, Hong JR, Yang SM, Yoo HM, Park DS (2009). Immediate implant placement into infected and noninfected extraction sockets: A pilot study. Oral Surg Oral Med Oral Pathol Oral Radiol Endod.

[25] Waasdorp JA, Evian CI, Mandracchia M (2010). Immediate placement of implants into infected sites: a systematic review of the literature. J Periodontol.

[26] ViskiÄ J, MilardoviÄ S, Katanec D, VojvodiÄ D, MehuliÄ K (2011). Immediate implantation in infected tooth sockets. Coll Antropol.

[27] Bell CL, Diehl D, Bell BM, Bell RE (2011). The immediate placement of dental implants into extraction sites with periapical lesions: a retrospective chart review. J Oral Maxillofac Surg.

[28] Corbella S, Taschieri S, Tsesis I, Massimo DF Postextraction implant in sites with endodontic infection as an alternative to endodontic retreatment: a review of literature. J Oral Implantol.

[29] Marconcini S, Barone A, Gelpi F, Briguglio F, Covani U Immediate Implant Placement in Infected Sites: A Case Series. J Periodontol.

